# A Predictive Model to Determine the Pattern of Nodal Metastasis in Oral Squamous Cell Carcinoma

**DOI:** 10.1155/2018/8925818

**Published:** 2018-05-13

**Authors:** B. S. M. S. Siriwardena, I. K. Rambukewela, T. N. Pitakotuwage, M. N. G. P. K. Udagama, P. V. R. Kumarasiri, W. M. Tilakaratne

**Affiliations:** ^1^Department of Oral Pathology, Faculty of Dental Sciences, University of Peradeniya, Peradeniya, Sri Lanka; ^2^Department of Community Medicine, Faculty of Medicine, University of Peradeniya, Peradeniya, Sri Lanka

## Abstract

**Background:**

Developing histological prediction models that estimate the probability of developing metastatic deposit will help clinicians to identify individuals who need either radical or prophylactic neck dissection, which leads to better prognosis. Identification of accurate predictive models in oral cancer is important to overcome extensive prophylactic surgical management for neck nodes. Therefore, accurate prediction of metastasis in oral cancer would have an immediate clinical impact, especially to avoid unnecessary radical treatment of patients who are at a low risk of metastasis.

**Methods:**

Histologically confirmed OSCC cases with neck dissection were used. Interrelation of demographic, clinical, and histological data was done using univariate and multivariate analysis.

**Results:**

465 cases were used and presence of metastasis and extracapsular invasion were statistically well correlated with level of differentiation (*p* < 0.001) and pattern of invasion (*p* < 0.001). Multivariate analysis showed level of differentiation, pattern of invasion, and stage as predictors of metastasis.

**Conclusions:**

The proposed predictive model may provide some guidance for maxillofacial surgeons to decide the appropriate treatment plan for OSCC, especially in developing countries. This model appears to be reliable and simple and may guide surgeons in planning surgical management of neck nodes.

## 1. Introduction

Developing histological prediction models that estimate the probability of developing metastatic deposit will help clinicians to draw effective treatment plans. That allows the clinician to identify individuals who need either radical or prophylactic neck dissection, which prevents unnecessary undertreatment or overtreatment leading to better prognosis. Oral squamous cell carcinoma (OSCC) is the most frequent malignant tumour of the head and neck region, representing the sixth leading cancer by incidence, and 500000 new cases are reported every year worldwide [1]. In Sri Lanka, it is the commonest cancer among men [2]. Majority of OSCC patients seek treatment at the advanced stage of the disease with subsequent poor prognosis.

Various studies using different statistical models have struggled to predict patients' metastasis and survival by analyzing the relationships between clinicopathological data and biomarkers (either newly developed or existing ones). However, it is difficult to find out practically successful biomarkers as the patients' survival is related to multiple factors. Therefore, current research direction is to identify various histological characteristics of the tumour which predict prognosis.

Studies indicated that histological tumour differentiation and lymph node metastasis [3] could be good predictors when designing therapeutic strategies for OSCC. Therefore, it is worthwhile to evaluate these potential biological properties and provide predictive information of behavior of the cancer preoperatively.

We have shown with our previous studies that pattern of invasion at the advancing front of the tumour and level of differentiation are some of the individual histological parameters that help to predict regional lymph node metastasis [4, 5]. Apart from histological parameters, tumour staging is one of the commonly used models when deciding the treatment plan [6].

Despite the fact that significant advances were achieved in surgery and chemotherapy over the past years, OSCC still shows a poor prognosis and lower survival rates [5, 7, 8]. If we have a model that predicts the behavior of the tumour using histopathological characteristics in combination with tumour stage, clinician is in a position to customize their treatment plan which will enhance the survival.

Therefore, we tried to develop a model using clinical and histopathological parameters to predict nodal status in OSCC, as it is difficult to incorporate molecular investigations in daily reporting practices due to lack of facilities in the developing parts of the world, where oral cancer's incidence is much higher.

## 2. Materials and Methods

We used data from the database of the country's only Head and Neck Pathology Centre, Faculty of Dental Sciences, University of Peradeniya, Sri Lanka. In the present study, we included all the patients with histologically confirmed OSCC who had undergone surgical resection with neck dissection from 1999 to 2012. The tumours have been staged according to TNM classification of UICC (Union for International Cancer Control) [9]. Ethical clearance for the study was obtained from the Faculty Research and Ethical Review Committee (certificate of ethical clearance, number FDS-FRC/2013/01). Informed consent was obtained from all individual participants included in the study before the surgical procedure.

Data in relation to age at first diagnosis, gender, subsites of cancer (buccal mucosa, tongue, upper alveolar ridge, lower alveolar ridge, palate, and floor of the mouth), and clinical stage were collected from patient records. Based on the histopathological reports, pattern of invasion (pattern I, large islands; pattern II, small islands; pattern III, thin strands; and pattern IV, individual cells), level of tumour differentiation (well-differentiated squamous cell carcinoma, moderately differentiated squamous cell carcinoma, or poorly differentiated squamous cell carcinoma), and nodal status (whether the node is positive or negative for tumour and the presence or absence of extracapsular spread in each ode in each level) were recorded. Tumour differentiation was performed according to Bryne and Anneroth's grading criteria that were described in one of our previous studies [5]. The whole group was further divided into young (<40 years) and old (>41 years) groups. This was based on the available literature including our own work [5–7]. Patients with inadequate data such as age gender site and histological materials were excluded from the study. Due to inadequate numbers to perform statistical analysis, tumours from lip, commissure, and retromolar region and histological subtypes such as basaloid, adenoid, adenosquamous, and spindle cell squamous cell carcinomas were also excluded from the study.

Univariate analysis was performed between each two characteristics. Chi Square test was also performed. Multivariate logistic regression was performed to develop the predictive model to identify the level of nodal metastasis. Data were analyzed using* SPSS* predictive analytics* software* version 11 for windows.

## 3. Results

A total of 465 cases with all necessary data were selected for the study. There were 329 males and 136 females with a M : F ratio of 2.6 : 1. The age ranges from 24 to 86 years with a mean age of 57.8 years. More than 50% of the cases were in the age group of 50–70 years at the time of diagnosis and majority of them were males. Buccal mucosa (37.2%) and tongue (24.1%) were the dominant subsites of OSCC in this study (Table 1). Tumour staging is one of the most important factors to predict prognosis. Although documented data is sparse in Sri Lanka, most OSCC patients seek treatment usually in the late stage of the disease. In this study, it was revealed that 43.5% and 32.8% of the patients presented to clinicians at stages 4 and 3, respectively. Well-differentiated and moderately differentiated tumours were significantly common compared to poorly differentiated cancers that represented only 7% of the total sample (Table 2).

The sample was divided into two groups depending on the age as young (<40 years) and old (>40 years). In the present study, more than 95% of the patients were above 40 years of age, which shows that OSCC is mostly a disease of the elderly. Most of the younger group had moderately differentiated tumours compared to the older group who had equal numbers of well-differentiated and moderately differentiated cancers (Table 1). It is interesting to note that the majority of young patients did not have associated habits and the common sites recorded were tongue and floor of the mouth. In the patients with the history of betel chewing habit, commonly affected site was buccal mucosa followed by lower alveolar ridge irrespective of the age.

**Figure 1:**
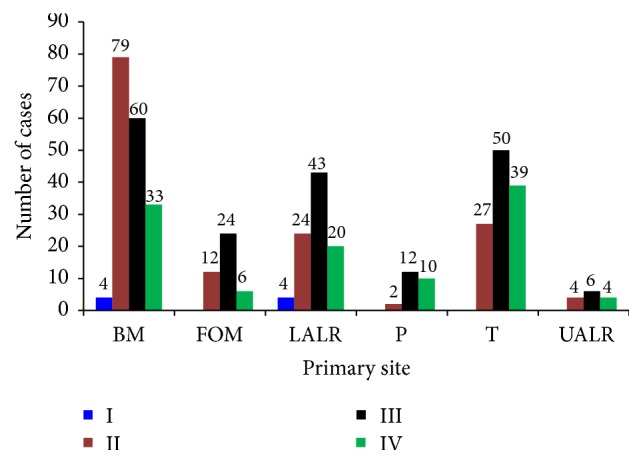
BM: buccal mucosa, FOM: floor of the mouth, LALR: lower alveolar ridge, P: palate, T: tongue, UALR: upper alveolar ridge. I, II, III, and IV: pattern of invasion types at the advancing front of the tumour.

Pattern of invasion has an impact on prognosis and was first described by Jakobsson et al. [8] in 1973. We have shown the reliability of predicting metastasis with pattern of invasion in our previous studies [5, 9]. The previous results can be further confirmed with the present study as the sample size is much larger. The present study found the majority of tongue, floor of the mouth, and lower alveolar ridge tumours to have invasive pattern III compared to other sites (Table 2 and Figure 1). Pattern of invasion and metastasis showed statistically significant association (*p* < 0.001) with patterns III and IV revealing higher metastatic rates compared to patterns I and II (Table 2). The relationship between clinicopathological factors and nodal metastases is summarized in Table 2. Presence of metastasis and extracapsular invasion were statistically well correlated with level of differentiation (*p* < 0.001) and pattern of invasion (*p* < 0.001).

We used a multivariate logistic regression model to identify the predictive factors of metastasis. This was done using the following variables: invasive pattern, level of differentiation, and tumour stage (Table 3). Level of nodal positivity (levels I–V) was taken as the dependent variable. Although these parameters were proven individually as helpful parameters to plan treatment, there are no documented studies combining these factors to describe a predictive model. We have tried to describe a predictive model using regression analysis in relation to the primary sites of buccal mucosa (Table 4) and tongue. Due to inadequate numbers, only buccal mucosa was considered for the predictive model. A few possible treatment plans for different levels of parameters should be as follows according to the prediction model. It is shown that if a buccal mucosal tumour is in stage 1 (T1N0) or 2 (T2N0) and histological pattern of invasion III or IV predicted nodal positivity goes up to cervical nodal level 3 (Table 4), then the proposed surgical treatment plan should be a selective neck dissection including levels I, II, and III as the minimum. Similarly, for stage 4 buccal cancers with infiltrating pattern IV, the treatment plan should be removal of cervical lymph nodes up to level 5. Further, to elaborate the model, if tumour shows invasive pattern I but stage 3, only up to level I clearance is adequate. Therefore, the proposed predictive model may provide some guidance for maxillofacial surgeons to decide treatment plan, especially in developing countries, where sophisticated diagnostic tools are least available. The number of cases was inadequate to perform the same for other subsites.

## 4. Discussion

The prognosis of patients with OSCC is directly related to the presence of lymph node metastasis [9]. A significant percentage of patients with early stages of OSCC have a poor prognosis despite the small size of the tumour [10, 11]. Hence, TNM staging system used in clinical practice does not provide information on the biological characteristics and aggressive clinical behavior of oral SCC.

However, the relationship between prognosis and clinicopathological features of OSCC has not been fully explored in Sri Lankan patients. If it is possible to elucidate potential clinicopathological parameters or predictors of nodal metastasis, they could be used to identify patients for management of neck nodes. Therefore, accurate prediction of metastasis in OSCC would have an immediate clinical impact, especially to avoid unnecessary radical treatment of patients leading to severe morbidity, despite the fact that they are at a low risk of metastasis.

Oral squamous cell carcinoma displays a wide range of metastatic behaviors that cannot be predicted by a single feature alone such as tumour size, histology, or even individual gene or protein expression/activity. Furthermore, the presence or absence of nodal metastasis consistently shows a statistical correlation with survival [12–16]. Metastasis is a process whereby genetic instability of the primary tumour fuels cell heterogeneity, allowing for a few tumour cells to disseminate cancer at distance [14]. Molecular markers such as HER-2 in breast cancer and chromosomal translocation in lymphomas are useful in planning treatment of above malignancies. However, to date, no molecular marker is used in therapeutic decision-making in OSCC.

In our previous studies, we have shown that there is a significant expression of molecular markers, hypoxia inducible factor-1*α* (HIF-1*α*) [17, 18], periostin [19], Matrix Metalloproteinase-9 (MMP-9) [20], Vascular Endothelial Growth Factor-C (VEGF-C) [21, 22], epidermal growth factor receptor (EGFR) [23], and *β*-Catenin [23], in OSCCs, as well as their ability to serve as potential molecules for predicting prognosis and metastasis. Further, it has been stated that the use of molecular markers together with the traditional histological methods may improve the strategy for comprehensive management of patients with OSCC [24]. This may provide a better evaluation of prognosis and the essentiality of elective neck dissection, which on the contrary may reduce the morbidity and cost of the advanced procedures. Further, Cyclin D1 is also positively correlated with nodal metastasis and poorer survival [25].

Although the significance of many of the biomarkers in OSCC has been reported, large prospective studies integrating the most reliable predictive biomarkers for OSCC are crucial to control the variability and for definite clarification of the prognostic significance of some of these molecular markers [24]. However, the objective of the present study is to propose a predictive model using clinical and histopathological parameters, as most centres in this part of the world have no facilities for molecular diagnostic techniques.

Head and neck surgery is generally considered to be challenging because of complex anatomy with difficulties of access and due to the presence of multiple vital structures. Most of the OSCC patients in this part of the world present to the clinicians at late stages of the disease. These advanced tumours are frequently treated with surgery followed by concurrent chemotherapy and radiation therapy. Neck dissections are associated with significant morbidity, which discourages the use of elective neck dissections. Therefore, a reliable method is needed to predict lymph node metastasis. The mortality and morbidity depend on the extent of surgical intervention. Radical neck dissection invariably leads to significant functional and cosmetic morbidity. Therefore, proper treatment planning is mandatory [26].

Recent advances have been used in the diagnosis and treatment of OSCC including identification of plasma or serum biomarkers, imaging techniques, biological therapy, and surgical techniques. However, these techniques have a little effect on overall survival of cancer [27, 28]. Technologies in functional genomics such as microarray analysis permit qualitative and quantitative biologic investigations on genome-wide scale [29], which is very expensive and cannot be practiced in frequent day-to-day reporting. Therefore, in this study, we were able to elucidate a combination of some histopathological parameters and clinical criteria to predict metastasis.

Detection of cervical lymph node metastasis in OSCC is of utmost importance in terms of surgical planning and prognosis. In this study, we reconfirmed that the pattern of invasion is the most important histological parameter associated with cervical lymph node metastasis. This was supported by other studies showing that patterns III and IV at the invasive front had higher incidence of neck metastasis and poor prognosis than those who had patterns I and II [4, 5]. Degree of tumour differentiation has also been found to correlate with nodal metastasis in OSCC [30]. Our study also confirms the above statement that poorly differentiated tumours show higher metastatic rates.

Tumours involving palate and upper alveolar ridge showed higher metastatic rates compared to other intraoral sites. Although some clinical parameters such as age, gender, and primary site did not reach statistical significance with metastasis, there was an increased tendency of extracapsular invasion in relation to younger patients, female patients, and sites such as tongue, palate, and upper alveolar ridge. There was a positive correlation between pattern of invasion and extracapsular invasion. Similarly, the same relationship was found between stage and extracapsular invasion. Therefore, this information may help to determine the extent of neck dissection and reduce the morbidity associated with unnecessary surgery or irradiation. It has been shown that tumour stage has a marked correlation with prognosis of OSCC [5]. The present study also proved that the stage of a given OSCC is a major factor in predicting metastasis.

Statistical models have always been a helpful tool in modeling disease susceptibility prediction. Logistic regression is a mathematical modeling approach that is used to describe the relationship between several explanatory variables and a dichotomous dependent variable. By using logistic regression, one can predict the outcome from a set of variables that may be continuous, discrete, and dichotomous or a mix of any of these [31]. Predictive regression models characterize the relationship between inputs and outputs using linear equation for linear functions. Interpretations of the logistic regression conclude that pattern of invasion, level of differentiation, and tumour stage have positive contribution to metastatic susceptibility as shown in Table 3. The criteria used for ultimate prediction is the pattern of invasion and tumour stage (TNM), which can be used as a guide to predict nodal metastasis. In the present study, it is clearly indicated that invasive pattern and stage can predict the level of metastasis. Further, for example, if tumour shows invasive pattern I but stage 3, only up to level I clearance is adequate. Similarly, if the tumour is in stage 4 with invasive pattern IV, neck clearance up to level 5 is indicated.

Models based on multiple variables that predict outcome in terms of lymph node metastasis seem to be more accurate than prediction based on single variables [32]. A system proposed by Woolgar and Scott [33] showed that tumour thickness and expression of factor VIII in combination predict nodal metastasis and local recurrence. Similarly, pattern of invasion and immunohistochemical expression of periostin and VEGF-C were also significantly correlated with metastasis [21].

## 5. Conclusion

Identification of accurate predictive models in OSCC is important to overcome extensive prophylactic surgical management for neck nodes. The therapeutic regimen should be judged by means of not only T classification or stage of the tumour but also evaluation on the histological parameters such as invasive pattern and level of differentiation. In the present study, the proposed predictive model appears to be reliable and simple and may guide the surgeon in planning surgical management of neck nodes for OSCC. Further studies are necessary to identify predictive models in other sites of the oral cavity.

## Figures and Tables

**Table 1:** Relationship of tumour differentiation with age, sex, and tumour site.

	Well-differentiated SCC (%)	Moderately differentiated SCC (%)	Poorly differentiated SCC (%)	Total
Age groups				
20–40	9 (45)	11 (50)	1 (5)	21
41–50	34 (38)	49 (55)	6 (7)	89
51–60	88 (50)	70 (40)	18 (10)	176
61–70	71 (53)	57 (42)	7 (5)	135
71–86	23 (52)	19 (43)	2 (6)	44

Gender				
Male	153	153	23	329
Female	72	53	11	136

Primary site				
BM	104 (60)	58 (33.5)	11 (6.4)	173
FOM	19 (44.2)	24 (55.8)	0 (0)	43
LALR	47 (50.5)	44 (47.3)	2 (2.2)	93
P	6 (25)	11 (45.8)	7 (29)	24
T	43 (38.4)	57 (50.9)	12 (10.7)	112
UALR	6 (30)	12 (60)	2 (10)	20

BM: buccal mucosa, FOM: floor of the mouth, LALR: lower alveolar ridge, P: palate, T: tongue, UALR: upper alveolar ridge.

**Table 2:** The relationship between clinicopathological parameters and nodal status in patients with OSCC.

Variable	Frequency (%)	Positive nodes (%)	Extra capsular invasion (%)	*p* value^*∗*^
Age				
20–40	21 (4.5)	11 (52.3)	11	NS
41–50	90 (14.35)	42 (46.6)	31	
51–60	177 (38.06)	82 (46.3)	60	
61–70	133 (28.6)	54 (40.6)	40	
71–86	44 (9.4)	19 (43.18)	17	

Gender				
Male	329 (69.8)	149 (45.3)	110	NS
Female	136 (29.2)	60 (44.1)	50	

Primary site				
BM	173 (37.2)	66 (38.15)	53	NS
FOM	43 (9.2)	19 (4.1)	12	
LLR	93 (20)	39 (41.9)	28	
P	24 (5.2)	21 (87.5)	19	
T	112 (24.1)	52 (46.4)	39	
UALR	20 (4.3)	12 (60)	9	

Tumour stage				
1	17 (5.68)	0 (0)	0	0.001
2	54 (18.06)	1 (1.8)	1	
3	98 (32.77)	49 (50)	30	
4	130 (43.47)	87 (66.9)	78	

Tumour differentiation				
Well	225 (48.4)	51 (26.7)	36	0.001
Moderate	206 (44.3)	124 (60.2)	92	
Poor	34 (7.3)	23 (67.7)	20	

Invasive front				
Inv. I	7 (1.5)	0 (0)	0	0.001
Inv. II	142 (30.53)	28 (19.7)	17	
Inv. III	200 (43.01)	102 (51)	76	
Inv. IV	113 (24.3)	79 (69.91)	67	

^*∗*^Chi-square test. NS: not significant.

**Table 3:** The final regression model identifies differentiation, invasion, and stage as the predictors of the level of positivity in OSCC.

*Coefficients* ^a^						
Model		Unstandardized coefficients		Standardized coefficients	*t*	Sig.
		*B*	Std. error	Beta		
(1)	(Constant)	−1.182	.231		−5.123	.000
	stage	.711	.071	.505	10.071	.000

(2)	(Constant)	−2.605	.293		−8.902	.000
	Stage	.620	.067	.440	9.320	.000
	Invasion	.573	.081	.336	7.113	.000

(3)	*(Constant)*	−2.642	.291		−9.081	.000
	*Stage*	.611	.066	.434	9.226	.000
	*Invasion*	.446	.097	.261	4.600	.000
	*Differentia*	.268	.116	.131	2.322	.021

^a^Dependent variable: level of nodal positivity. Adjusted *R*-squared = 0.368; the final regression model (3).

**Table 4:** A model developed to predict lymph node metastasis in patients with oral squamous cell carcinoma of buccal mucosa.

	Level 1	Level 2	Level 3	Level 4	Level 5
IP 1 + S 3	√				
IP 2 + S 1	√				
IP 2 + S 2	√				

IP 1 + S 4		√			
IP 2 + S 3		√			
IP 3 + S 1		√			
IP 3 + S 2		√			

IP 2 + S 4			√		
IP 3 + S 3			√		
IP 4 + S 1			√		
IP 4 + S 2			√		

IP 3 + S 4				√	
IP 4 + S 3				√	

IP 4 + S 4					√

IP: pattern of invasion; S: stage of the tumour.
